# Improvement of fatigue in generalised myasthenia gravis with zilucoplan

**DOI:** 10.1007/s00415-024-12209-3

**Published:** 2024-02-24

**Authors:** Michael D. Weiss, Miriam Freimer, M. Isabel Leite, Angelina Maniaol, Kimiaki Utsugisawa, Jos Bloemers, Babak Boroojerdi, Emily Howard, Natasa Savic, James F. Howard

**Affiliations:** 1 Department of Neurology, University of Washington Medical Center, Seattle, WA USA; 2 Department of Neurology, The Ohio State University Wexner Medical Center, Columbus, OH USA; 3 Nuffield Department of Clinical Neurosciences, University of Oxford, Oxford, UK; 4 Department of Neurology, Oslo University Hospital, Oslo, Norway; 5 Department of Neurology, Hanamaki General Hospital, Hanamaki, Japan; 6 UCB Pharma, Brussels, Belgium; 7 UCB Pharma, Monheim, Germany; 8 UCB Pharma, Slough, UK; 9 Cogent Skills, Warrington, UK; 10 UCB Pharma, Bulle, Switzerland; 11 Department of Neurology, The University of North Carolina at Chapel Hill, Chapel Hill, NC USA; 12 Present Address: University of Bath, Bath, UK

**Keywords:** Complement inhibitor, Fatigue, Myasthenia gravis, Quality of life, Zilucoplan

## Abstract

**Background:**

Fatigue is a debilitating symptom of myasthenia gravis (MG). The impact of fatigue on MG can be assessed by Quality of Life in Neurological Disorders (Neuro-QoL) Short Form Fatigue scale. Transformation of raw Neuro-QoL fatigue scores to T-scores is a known approach for facilitating clinical interpretation of clinically meaningful and fatigue severity thresholds.

**Methods:**

In the Phase 3, double-blind, placebo-controlled RAISE study (NCT04115293), adults with acetylcholine receptor autoantibody-positive generalised MG (MG Foundation of America Disease Class II–IV) were randomised 1:1 to daily subcutaneous zilucoplan 0.3 mg/kg or placebo for 12 weeks. Patients completing RAISE could opt to receive zilucoplan 0.3 mg/kg in an ongoing, open-label extension study, RAISE-XT (NCT04225871). In this post-hoc analysis, we evaluated the long-term effect of zilucoplan on fatigue in RAISE patients who entered RAISE-XT. We report change in Neuro-QoL Short Form Fatigue T-scores and fatigue severity levels from RAISE baseline to Week 60.

**Results:**

Mean Neuro-QoL Short Form Fatigue T-scores improved from baseline to Week 12 in the zilucoplan group (*n* = 86) with a clinically meaningful difference versus placebo (*n* = 88; least squares mean difference: − 3.61 (nominal *p*-value = 0.0060]), and these improvements continued further to Week 60. At Week 12, more patients on zilucoplan (*n* = 34, 47.2%) experienced improvements in ≥ 1 fatigue severity level from baseline versus placebo (*n* = 23, 28.4%; *p* = 0.017). At Week 60, most (*n* = 55, 65.5%) patients had mild fatigue or none.

**Conclusion:**

Treatment with zilucoplan demonstrated statistical and clinically meaningful improvements in fatigue scores and severity versus placebo during RAISE, which were sustained to Week 60 in RAISE-XT.

**Supplementary Information:**

The online version contains supplementary material available at 10.1007/s00415-024-12209-3.

## Introduction

Myasthenia gravis (MG) is a debilitating autoimmune neuromuscular disease characterised by fluctuating muscle weakness with a prevalence of 100–350 people per million globally [1, 2]. MG causes neuromuscular or muscle fatiguability, where patients’ symptoms worsen with sustained activity [3]. However, fatigue in MG goes beyond neuromuscular fatiguability and is also characterised by sensations ranging from tiredness to an overwhelming, debilitating and sustained sense of exhaustion that decreases patients’ capacity for physical, functional, social and mental activities [4, 5]. A patient-led analysis, with over 114 insights from patients with MG, identified fatigue as one of their major challenges. Patients articulated that fatigue can be debilitating, and unpredictable fluctuations in symptoms lead to feelings of vulnerability and lack of control [6].

According to a systematic review by Ruiter et al*.* across 21 studies, the prevalence of fatigue in patients with MG varied from 44 to 82% [7]. The authors suggest that the pathophysiology of fatigue is likely multifactorial in nature, and increased disease severity combined with reduced physical activity could result in lowered muscle strength and increased fatigue [7]. This correlation between disease severity and fatigue was further corroborated in a Dutch study of 420 patients, which found a statistically significant positive correlation between fatigue and MG severity [8]. Fatigue is also associated with depression [7]. As a result of all these factors, fatigue can have a major impact on the day-to-day lives of patients with MG and is strongly associated with reduced quality of life (QoL) [6, 9]. Patients describe MG as an “invisible illness” and may find it difficult to explain their challenges to others, which increases the feelings of frustration, loneliness and depression [6], and such feelings can be exacerbated by fatigue [5, 6, 9]. In a study of real-world clinical practice conducted in the United States, fatigue was included in the five most “troublesome” symptoms for patients with MG together with diplopia, ocular myasthenia, ptosis and weakness in the legs, as reported by physician-completed questionnaires [10]. In a recent study assessing daily activities of patients with MG, general fatigue was identified as one of the most salient symptoms, as reported by the patients [11]. Further, a qualitative, cross-sectional study in the United States with insights from 28 patients with generalised MG (gMG) reported that the most common patient-reported treatment goal for the management of their MG symptoms included reduced fatigue or weakness, followed by achieving symptom stability [12]. Moreover, as reported in another study of 73 patients with MG, treatment with corticosteroids did not alleviate the symptom of fatigue [13].

Clinical assessments of MG, such as Myasthenia Gravis Activities of Daily Living (MG-ADL) and Quantitative Myasthenia Gravis (QMG) scores, measure muscle weakness or fatiguability, but do not assess the general concept of fatigue [5]. Thus, more regular assessment and monitoring of fatigue as a symptom of MG is needed, as well as treatments that can improve it, in order to ultimately improve QoL for patients.

Fatigue can be assessed by a self-administered patient-reported outcome (PRO) measure known as the Quality of Life in Neurological Disorders (Neuro-QoL) fatigue scale [4]. This scale was developed as a disease-agnostic scale, and its development and validation have been performed in a range of disorders [5, 14–16]. In the Phase 3 study of zilucoplan in adult patients with gMG (RAISE; ClinicalTrials.gov identifier: NCT04115293), we used the Neuro-QoL Short Form Fatigue scale, a shortened version of the Neuro-QoL fatigue scale. Questions of the Short Form scale cover feelings of exhaustion, frustration at being unable to do tasks owing to fatigue, and limitations to social activity [17]. In addition, Neuro-QoL Short Form Fatigue raw scores can be converted into T-scores, for which clinically meaningful change and severity thresholds have been proposed. In 2015, Cook et al*.* determined severity thresholds for the Neuro-QoL Short Form Fatigue scale using an adapted bookmarking method with an expert panel of clinicians (*n* = 7) and people living with multiple sclerosis (MS; n = 6) [18]. Then, in 2017, Idio Scale Judgement methodology was used to calculate meaningful within-patient change (MWPC) thresholds, or the smallest change in a measure that is considered significant by patients, for Neuro-QoL Short Form Fatigue scores in MS, whereby changes from baseline of − 3.5 and + 3.2 indicated improvement and worsening, respectively [16]. Psychometric evidence supporting use of the Neuro-QoL Short Form Fatigue scale in MG was provided by Tran et al*.* in 2018, who also calculated the threshold for MWPC (individual-level minimal important difference) to be − 1.9 [5]. The improvement and worsening thresholds described above, based on Neuro-QoL Short Form Fatigue T-scores, can be applied to interpret between-group comparisons, within-patient (responder) comparisons, and the clinical meaningfulness of the fatigue measurements in our study [5, 16, 18].

Zilucoplan is a small macrocyclic peptide that targets the complement cascade by binding to complement component 5 (C5) with high affinity and specificity [19]. It works via a dual mechanism of action by preventing the cleavage of C5 to C5a and C5b, and also by hindering the formation of C5b6 should any C5b be formed, thereby blocking its binding to C6 and preventing the formation of membrane attack complex [20]. In the Phase 3 RAISE study, zilucoplan showed rapid, statistically significant and clinically meaningful improvements in MG-specific patient- and clinician-reported outcomes compared with placebo in a broad population of patients with acetylcholine receptor autoantibody-positive (AChR +) gMG [21]. In the exploratory endpoint of change from baseline (CFB) to Week 12 in Neuro-QoL Short Form Fatigue scores, there were statistically significant improvements in fatigue, reflected by the larger reduction in the raw scores of patients who received zilucoplan versus placebo, with a least squares (LS) mean difference (95% confidence interval [CI]) of − 3.06 (− 5.27, − 0.85; nominal p-value = 0.0069) [21].

Here, we report the effect of zilucoplan on fatigue in patients with AChR + gMG using T-scores from the Neuro-QoL Short Form Fatigue scale in RAISE and the ongoing open-label extension (OLE), RAISE-XT (ClinicalTrials.gov identifier: NCT04225871). A plain language summary of this study is available in Supplementary Text 1.

## Methods

### RAISE and RAISE-XT study designs

RAISE was a Phase 3, multicentre, randomised, double-blind, placebo-controlled study to assess the efficacy, safety and tolerability of zilucoplan in patients with gMG [21]. Patients self-administered subcutaneous doses of zilucoplan 0.3 mg/kg or placebo daily at home at approximately the same time each day for 12 weeks. RAISE-XT is an ongoing, multicentre, OLE study of zilucoplan where participants who completed the 12-week treatment period in either the Phase 2 double-blind study of zilucoplan [19] or RAISE could opt to receive daily subcutaneous doses of 0.3 mg/kg zilucoplan [22]. Full study designs of RAISE and RAISE-XT have been described elsewhere [21, 22].

### Study population

Full inclusion and exclusion criteria for RAISE have been described before [21]. Briefly, adults with AChR + mild-to-severe (Myasthenia Gravis Foundation of America [MGFA] Disease Class II–IV) gMG at screening were included [21]. All patients provided written informed consent.

### Assessments

Fatigue was assessed using the Neuro-QoL Short Form Fatigue scale at Weeks 1, 2, 4, 8 and 12 in the double-blind period and at Week 13 (Extension [E] Week 1), Week 14 (Week E2), Week 16 (Week E4), Week 20 (Week E8) and Week 24 (Week E12) in the OLE. After Week 24, Neuro-QoL Short Form Fatigue scores were assessed at quarterly visits. The Neuro-QoL Short Form Fatigue scale was self-administered and consisted of eight items with five Likert-type response options (“never” to “always”). Patients marked each item on a scale of 1–5 with a 7-day recall period [4, 17]. Raw total scores can range from 8 to 40, with higher scores indicating more severe fatigue [4, 17]. These Neuro-QoL Short Form Fatigue scale raw total scores at each visit were transformed into T-scores [4]. Change from double-blind baseline in Neuro-QoL Short Form Fatigue T-scores up to Week 60 in RAISE-XT was assessed post hoc, based on the pre-specified interim analysis cut-off date (8 September 2022). Additional efficacy assessments included the percentage of Neuro-QoL Short Form Fatigue T-score responders at Week 12 (also defined using the MWPC threshold of − 3.5), and transition between fatigue severity status from the baseline of double-blind studies to Week 12 and Week 60. Safety was mainly assessed by the incidence of treatment-emergent adverse events (TEAEs).

### Statistical design

Neuro-QoL Short Form Fatigue raw scores were transformed into T-scores with a mean of 50 and a standard deviation of 10 [4] with a range of 29.5–74.1, based on a clinical reference population [16]. A normal distribution is not required to use the Neuro-QoL Short Form Fatigue T-score distribution, as these scores are intended to be used to interpret the score of an individual. A clinically meaningful improvement from baseline was defined as a change of − 3.5, and clinically meaningful worsening was defined as a change of + 3.2, in Neuro-QoL Short Form Fatigue T-score from double-blind baseline [16]. This between-group analysis was interpreted based on the MWPC thresholds as determined by Cook et al. [16] and, as between-group meaningful change thresholds are typically lower than MWPC thresholds [23], these thresholds were used to allow for a more conservative approach instead of the threshold of − 1.9 as defined in an MG sample [5]. Responders were defined as patients who achieved clinically meaningful CFB (a greater than − 3.5 change) [16]. The fatigue severity level thresholds for T-scores used in this study have previously been defined as: none (“no problems”), < 45; “mild problems”, 45–55; “moderate problems”, 56–65; and “severe problems”, > 65 [18], which have also been used in an MG study [5]. The Neuro-QoL Analysis Set is defined as all randomised RAISE study participants who had a Neuro-QoL Short Form Fatigue score at baseline. As fatigue was not assessed in the Phase 2 study, patients who transitioned into RAISE-XT from the Phase 2 study are not included in this analysis.

For the responder analysis, odds ratios were calculated using the Cochran–Mantel–Haenszel (CMH) test with no stratification variables. P-values for the comparison of treatment groups are based on the CMH test from the general association and were nominal. Correlations were measured using Pearson’s correlation coefficient and Spearman’s correlation coefficient.

### Psychometric properties

Construct validity for use of the Neuro-QoL Short Form Fatigue scale in MG was assessed by investigating known-groups validity, in addition to convergent and divergent validity. A known-groups validity analysis was conducted to investigate the ability of the Neuro-QoL Short Form Fatigue scale to differentiate between groups that are expected to differ on this instrument, using MGFA disease classification scores at screening. The correlation between Neuro-QoL Short Form Fatigue T-scores and MG-ADL, QMG and Myasthenia Gravis Quality of Life 15-item revised (MG-QoL 15r) scores was studied for assessing convergent and divergent validity. MG-ADL, QMG and MG-QoL 15r scores used to analyse the psychometric properties of the Neuro-QoL Short Form Fatigue scores are described in Supplementary Text 2.

## Results

### Patients

In RAISE, 174 patients were randomised to receive zilucoplan 0.3 mg/kg (*n* = 86) or placebo (*n* = 88). After Week 12, all patients who completed RAISE (166 [95.4%]) entered RAISE-XT to receive zilucoplan 0.3 mg/kg and were included in this analysis. Their baseline characteristics are described elsewhere [21]. Overall, a broad gMG population was included; patients had mild-to-severe gMG according to the MGFA disease classification criteria. Median (range) exposure to zilucoplan in the OLE for the patients who entered from both Phase 2 and Phase 3 (RAISE) studies (*N* = 200), the population used in the safety analysis, was 1.2 (0.11–4.45) years. At the data cut-off, of all patients enrolling from either the Phase 2 or Phase 3 study, 166 (83.0%) patients were continuing to receive zilucoplan in the RAISE-XT study, and 34 (17.0%) patients discontinued the study. The most common reason for discontinuation was voluntary withdrawal by the patient (12 [6.0%]).

### Change from baseline in Neuro-QoL Short Form Fatigue T-scores

**Fig. 1:**
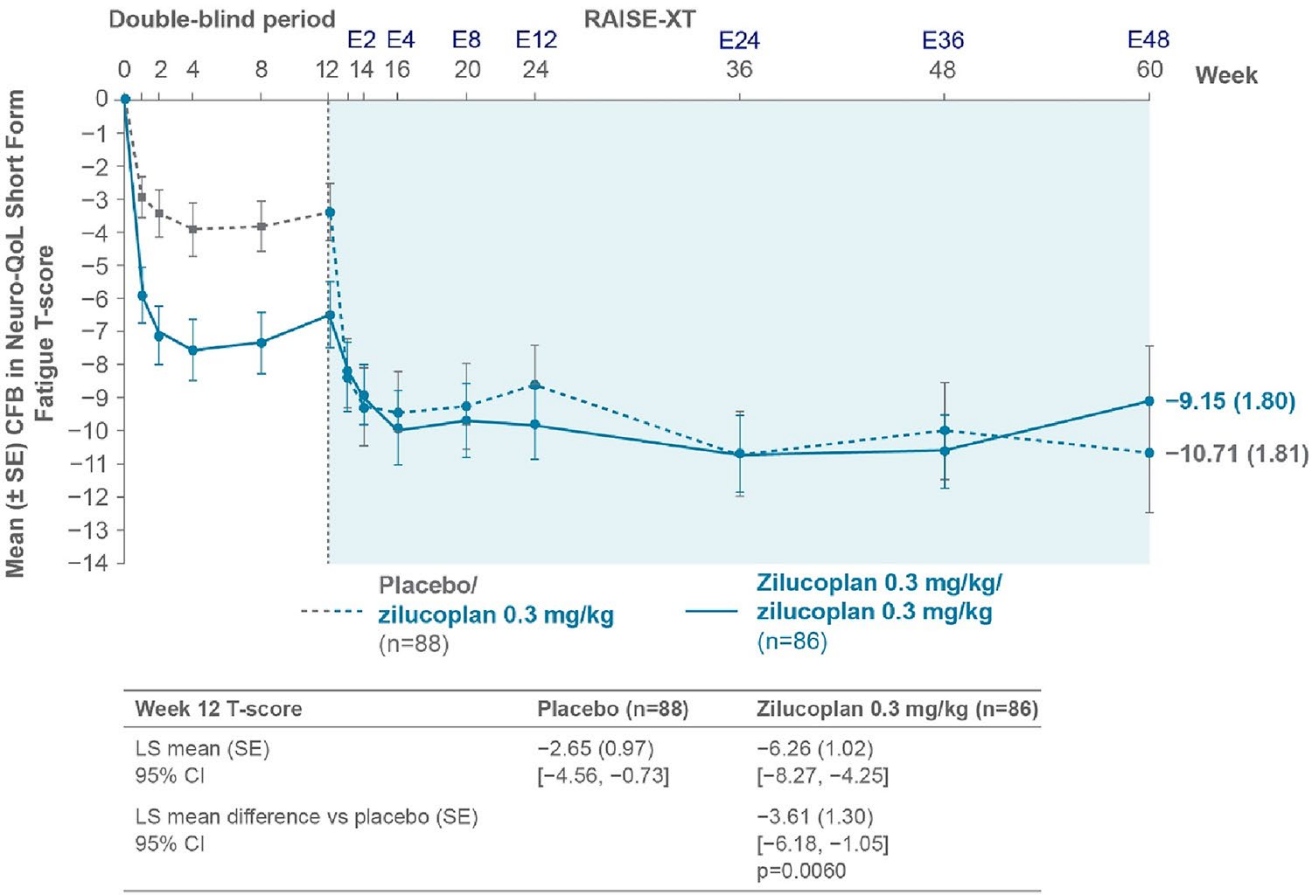
Change from double-blind baseline to Week 60 in Neuro-QoL Short Form Fatigue T-scores. This analysis included only patients from the Phase 3 RAISE parent study. Statistical analysis on Week 12 Neuro-QoL Short Form Fatigue T-scores was performed using the mITT population in RAISE. *CFB* change from baseline, *CI* confidence interval, *E* extension, *LS* least squares, *mITT* modified intent-to-treat, *Neuro-QoL* Quality of Life in Neurological Disorders, *SE* standard error.

During RAISE, in the zilucoplan group, mean Neuro-QoL Short Form Fatigue T-scores improved from double-blind baseline to Week 12, as compared with placebo, with an LS mean difference of − 3.61 (nominal *p*-value = 0.0060; Fig. 1). This between-group difference in CFB is considered clinically meaningful as it exceeds the MWPC threshold of − 3.5. During RAISE-XT, these scores continued to improve further through to Week 24, with a mean [standard error (SE)] CFB of − 9.81 (1.16) for the zilucoplan group, and were sustained through to Week 60 [mean (SE) CFB − 9.15 (1.80)]. Among patients who transitioned from placebo to zilucoplan in the OLE (placebo-switch group), rapid improvements were observed at the first week after switching to zilucoplan 0.3 mg/kg (Week 13) and further improvements were observed through to Week 24, with a mean (SE) CFB of − 8.63 (1.24); these improvements were sustained through to Week 60 with a [mean (SE) CFB − 10.71 (1.81)] (Fig. 1).

### Neuro-QoL Short Form Fatigue T-score responder analysis at Week 12

**Fig. 2:**
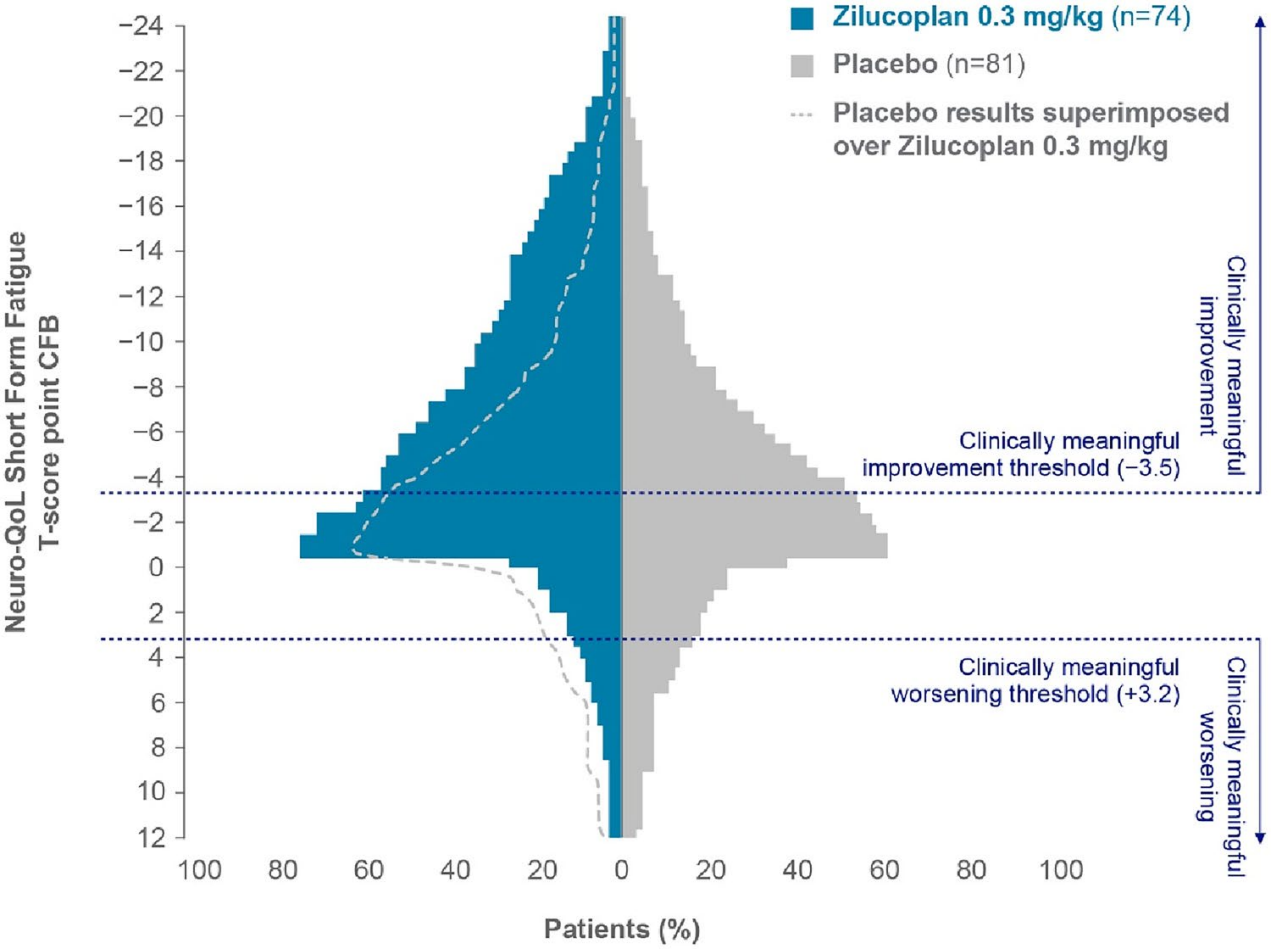
Responder analysis in Neuro-QoL Short Form Fatigue T-scores at Week 12 in RAISE. The analysis was performed using the Neuro-QoL Analysis Set, which is defined as all patients who had a non-missing Neuro-QoL Short Form Fatigue T-score at RAISE baseline. For the clinically meaningful improvement threshold of − 3.5, the OR (95% CI) versus placebo was 1.15 (0.61, 2.17) with a nominal *p*-value of 0.659. For the clinically meaningful worsening threshold of + 3.2, the OR (95% CI) versus placebo was 0.66 (0.27, 1.64) with a nominal *p*-value of 0.372. *CFB* change from baseline, *CI* confidence interval, *Neuro-QoL* Quality of Life in Neurological Disorders, *OR* odds ratio

More patients on zilucoplan had reductions in fatigue from baseline versus placebo at the end of the double-blind period (Week 12; Fig. 2). A higher proportion of patients on zilucoplan reached the clinically meaningful improvement threshold (− 3.5), while a lower proportion of patients reached the clinically meaningful worsening threshold (+ 3.2) at Week 12 when compared with placebo.

### Fatigue severity transitions from double-blind study baseline

**Fig. 3:**
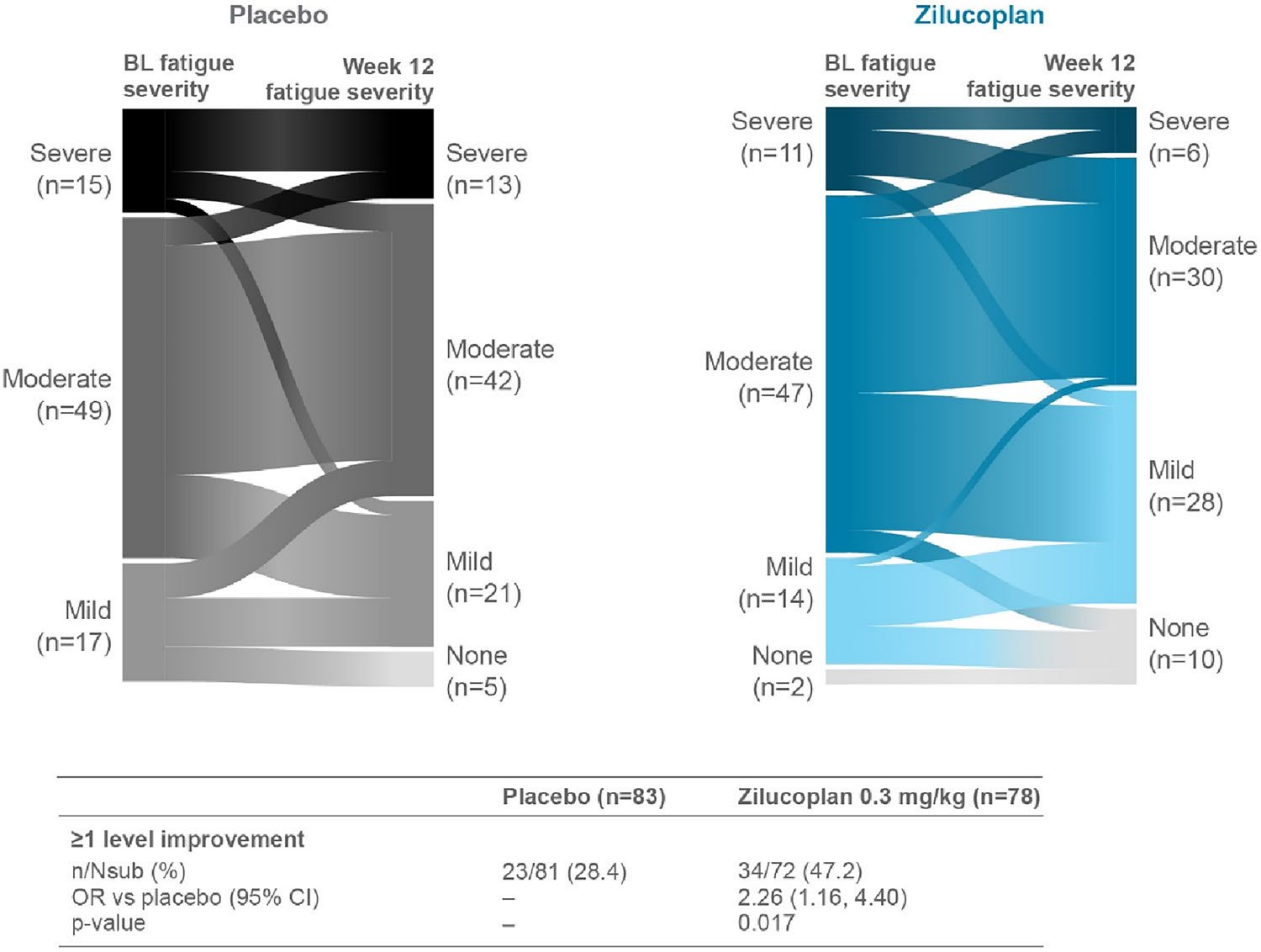
Fatigue severity level transition from double-blind baseline to Week 12. *n* = number of patients with at least one fatigue severity level improvement. *Nsub* number of patients with available information at the timepoint. Percentages are based on Nsub. OR calculated using CMH test with no stratification variables. Patients with “missing” Neuro-QoL Short Form Fatigue data at baseline excluded. The statistical analysis includes patients with baseline and Week 12 data who had a potential to improve (2 patients in the zilucoplan group reported “no problems” at baseline and were excluded from this analysis). *BL* baseline, *CI* confidence interval, *CMH* Cochran–Mantel–Haenszel, *OR* odds ratio

**Fig. 4:**
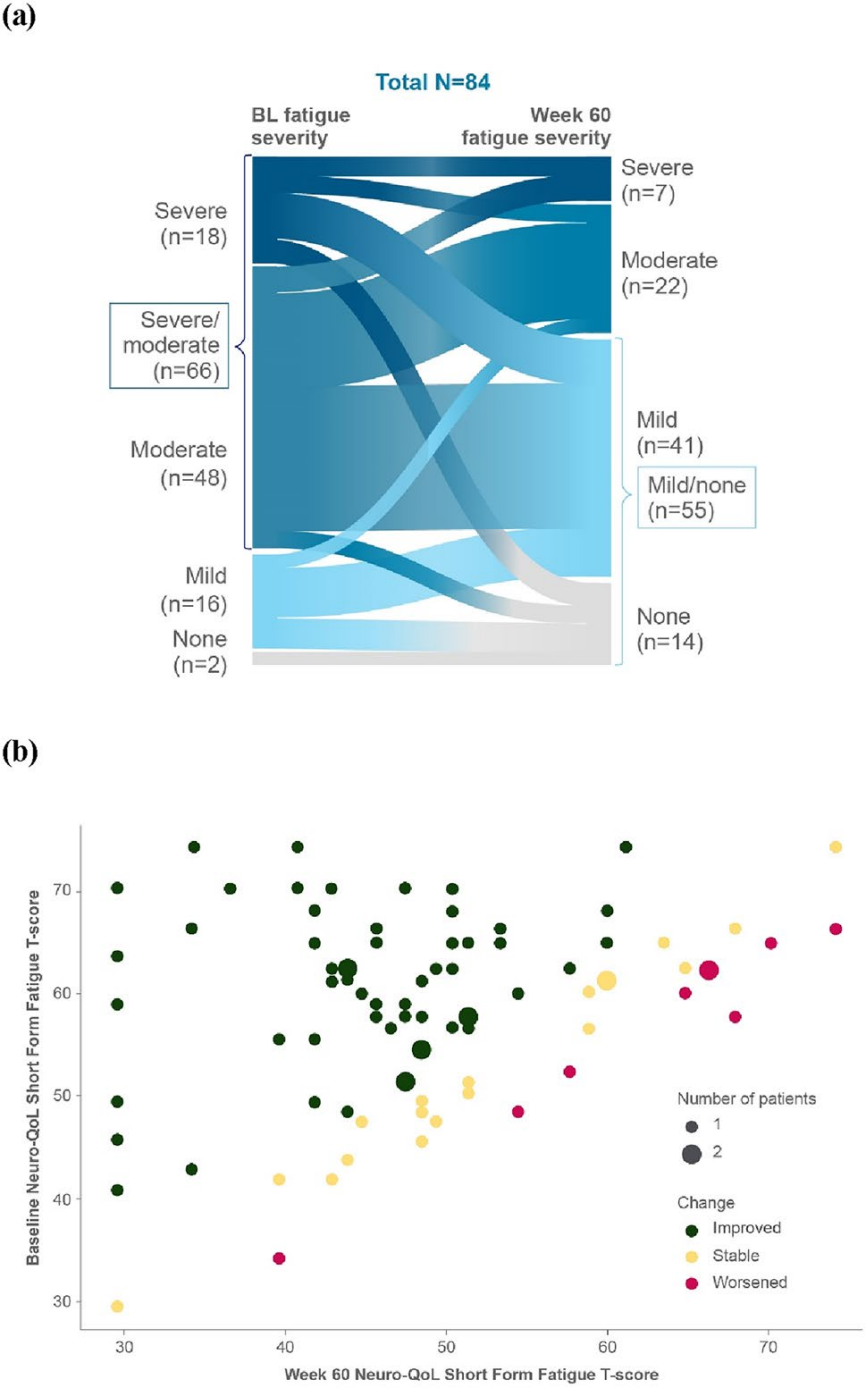
Fatigue severity level transition from double-blind baseline to Week 60 (**a**) and change in Neuro-QoL Short Form Fatigue T-scores at Week 60 vs double-blind baseline (**b**). The analysis set includes patients from RAISE and RAISE-XT who were included in the RAISE mITT population and had available Neuro-QoL Short Form Fatigue data at RAISE baseline and Week 60 (*N* = 84). “Improved” and “worsened” categorisation is based on the thresholds for clinically meaningful improvement (− 3.5) and clinically meaningful worsening (+ 3.2), respectively, in Neuro-QoL Short Form Fatigue T-scores from RAISE baseline. *BL* baseline, *mITT* modified intent-to-treat, *Neuro-QoL* Quality of Life in Neurological Disorders

At Week 12, more patients in the zilucoplan arm (*n* = 34, 47.2%) experienced improvements by one or more fatigue severity levels from baseline versus placebo (*n* = 23, 28.4%; nominal *p*-value = 0.017; Fig. 3). The improvements in Neuro-QoL fatigue severity levels observed during the double-blind period continued further into the OLE. At double-blind baseline, the majority of patients had “severe” or “moderate” fatigue (*n* = 66, 78.6%; *N* = 84; Fig. 4a). At Week 60, 8 of 18 patients with “severe” fatigue at double-blind baseline had transitioned to “mild” fatigue and another 4 patients with “severe” fatigue at baseline had no fatigue at Week 60. Additionally, at Week 60, 24 of 48 patients with “moderate” fatigue at double-blind baseline had transitioned to “mild” fatigue and another 3 patients with “moderate” fatigue at baseline had no fatigue at Week 60. Overall, at Week 60, the majority of patients had “mild” or no fatigue (*n* = 55, 65.5%; Fig. 4a). These findings were consistent across the zilucoplan group and in the placebo-switch group (data not shown). The majority of patients (*n* = 56, 66.7%; *N* = 84) at Week 60 experienced a clinically meaningful improvement in fatigue from baseline (Fig. 4b).

### Psychometric properties

There were positive correlations between Neuro-QoL Short Form Fatigue scale scores and MG-ADL, QMG and MG-QoL 15r scores at double-blind study baseline, Week 12 and Week 60 (*p* < 0.001 for all scores at all timepoints; Table 1). The correlation coefficients continued to increase as the study progressed and were highest at Week 60 for both MG-ADL and MG-QoL 15r scores. There was a highly positive correlation for MG-QoL 15r score and a moderately positive correlation for QMG and MG-ADL scores at baseline, which increased by Week 60. In the known-groups analysis, performed using MGFA disease classification scores at screening, differences in the mean Neuro-QoL Short Form Fatigue T-scores between patients categorised as MGFA Disease Class II versus MGFA Disease Class III/IV were not significant [− 2.16, 95% CI  − 5.03, 0.71; *p* = 0.140 (*F*-test); *p* = 0.092 (Kruskal–Wallis test); Supplementary Table 1].

**Table 1:** Correlation between change from double-blind baseline to Week 12 and Week 60 in Neuro-QoL Short Form Fatigue scores and MG-ADL, QMG and MG-QoL 15r scores

Visit	n	MG-ADL (*r*, *p*-value)		QMG (*r*, *p*-value)		MG-QoL 15r (*r*, *p*-value)	
		Pearson’s coefficient	Spearman’s coefficient	Pearson’s coefficient	Spearman’s coefficient	Pearson’s coefficient	Spearman’s coefficient
Baseline	161	0.485, < 0.001	0.462, < 0.001	0.433, < 0.001	0.447, < 0.001	0.771, < 0.001	0.771, < 0.001
Week 12	165	0.733, < 0.001	0.749, < 0.001	0.587, < 0.001	0.589, < 0.001	0.858, < 0.001	0.863, < 0.001
Week 60	92	0.770, < 0.001	0.775, < 0.001	0.609, < 0.001	0.576, < 0.001	0.890, < 0.001	0.897, < 0.001

*n* represents number of patients with available data for MG-ADL, QMG, MG-QoL 15r and Neuro-QoL Short Form fatigue scores at each time point

*MG-ADL* Myasthenia Gravis Activities of Daily Living, *MG-QoL 15r* Myasthenia Gravis Quality of Life 15-item revised, *Neuro-QoL* Quality of Life in Neurological Disorders, *QMG* Quantitative Myasthenia Gravis, *r* correlation coefficient

### Safety

Details of the safety analysis among the full RAISE-XT population have been described elsewhere [22]. Briefly, out of 200 enrolled patients, 188 (94.0%) patients experienced a TEAE, and 64 (32.0%) patients experienced serious TEAEs. The most common TEAEs were (worsening of) MG (26.0%), COVID-19 (24.5%), headache (17.5%), diarrhoea (15.0%) and nasopharyngitis (15.0%) [22]. Seventeen (8.5%) patients had a TEAE resulting in permanent withdrawal from treatment [22]. Overall, zilucoplan had a favourable safety profile in the long term and was well tolerated.

## Discussion

Fatigue is an important yet understudied symptom of MG [12]. To our knowledge, this is the first analysis of T-score transformation of Neuro-QoL Short Form Fatigue scores in patients with MG in a Phase 3 study, allowing us to assess the clinical meaningfulness of these data. Patients receiving zilucoplan in RAISE and its OLE experienced clinically meaningful improvements in fatigue compared with placebo, thus demonstrating a positive effect of zilucoplan on fatigue. The improvements in fatigue observed during the double-blind period of RAISE further continued into RAISE-XT, the ongoing OLE study, and were sustained up to Week 60, indicating that zilucoplan consistently improved fatigue in patients with MG in the long term. Most patients experienced moderate to severe fatigue at the start of the study, but patients on zilucoplan showed significant improvement in their fatigue severity at Week 12, with more patients on zilucoplan reporting clinically meaningful improvements in fatigue at Week 12 versus placebo. Furthermore, most of these patients with moderate or severe fatigue at baseline had no or mild fatigue after 60 weeks. RAISE-XT is ongoing, and at the time of the data cut-off, a large majority of patients were still enrolled. There may be a potential effect of study discontinuations on the efficacy data, however, most discontinuations were due to voluntary withdrawal and not due to a lack of efficacy.

This post-hoc analysis used T-scores, transformed from raw Neuro-QoL Short Form Fatigue data. Meaningful change thresholds, responder definitions, and severity thresholds have already been defined for T-scores and allow for an easier interpretation of the effect of change [5, 16, 18]. The improvements in fatigue observed here with T-score data were aligned with the improvements in raw scores observed in RAISE [21]. Using T-scores in this way provides us with a way to highlight clinically meaningful changes in fatigue in MG, which are important for the daily living of patients with MG, in addition to the clinically established measures such as MG-ADL and QMG scores. Another strength of this study is that the Neuro-QoL Short Form Fatigue scale was used, a validated, disease-agnostic, easy-to-use PRO measure with only eight items.

The known-groups validity analysis used MGFA disease classification scores at screening to assess whether the Neuro-QoL Short Form Fatigue scale differentiates between these groups. The mean fatigue T-scores for MGFA Disease Class II were lower than for the pooled MGFA Disease Class III/IV, but this difference was not statistically significant. This may be because the MGFA disease classification is subjective in nature, especially when it comes to distinction between classes [24], but in Tran et al., clear and significant differences were observed [5]. A limitation of our analysis is that it is possible that the inclusion criteria of our study, an MG-ADL score of ≥ 6 and a QMG score of ≥ 12 [21], resulted in more patients at the higher end of MGFA Disease Class II, making it harder to distinguish these patients from those in the pooled MGFA Disease Class III/IV. This explanation is supported by a higher mean T-score for MGFA Disease Class II in this study as compared with Tran et al.[5]. However, through the course of the OLE, Neuro-QoL Short Form Fatigue scores correlated positively with both the clinician-reported outcomes of QMG and the PROs of MG-ADL and MG-QoL 15r, highlighting the clinical relationship between MG-specific symptoms and fatigue. Fatigue is the most common patient-reported treatment goal for their MG [12], and therefore, should not be underestimated as a contributor to patients’ health-related QoL (HRQoL). It is important for clinicians to routinely measure fatigue as part of the overall assessment of MG symptoms in their patients, in addition to MG-specific outcomes.

Both Neuro-QoL Short Form Fatigue and MG-QoL 15r assess HRQoL and the impact MG has on patients’ lives; MG-QoL 15r is focused on MG more generally [25], while Neuro-QoL Short Form Fatigue is focused on the impact of fatigue specifically [17]. Both of these measures deal directly with the impact of MG on HRQoL and, as expected, there was a high correlation between them in our study at baseline, which further increased at Week 60. This high correlation further substantiates that a reduction in fatigue leads to an improvement in HRQoL in patients with MG. The items in the QMG scale focus on muscle fatiguability, with items that measure how long the patient can keep their arms outstretched at 90 degrees while sitting, legs at 45 degrees while supine, and hand grip [26]. Similarly, MG-ADL covers activities of daily living, such as impairment of ability to brush teeth or comb hair, and impairment of ability to rise from a chair [27]. While these symptoms do not necessarily reflect fatigue and are distinct concepts from those assessed by the Neuro-QoL Short Form Fatigue scale, there was a moderate, but statistically significant, positive correlation at baseline, supporting the known-groups validity analysis, which increased during the course of the study. The correlations at baseline indicate that there is a relationship between fatigue and muscle fatiguability, and the increase in correlations observed throughout the study is likely a result of the treatment effect of zilucoplan, causing patients, on average, to report improvement on all scales [21, 22].

The differences in magnitude of the correlations between Neuro-QoL Short Form Fatigue and MG-ADL, QMG, and MG-QoL 15r scores support the convergent and divergent validity of Neuro-QoL Short Form Fatigue in MG. The stronger correlations were observed with instruments assessing more comparable concepts (MG-QoL 15r assessing HRQoL) and less strong correlations with instruments assessing more distal concepts (MG-ADL assessing symptoms and the impact on daily activities, and QMG assessing muscle fatiguability). Taken together, the observed psychometric properties of the Neuro-QoL Short Form Fatigue scale in our study support its use in gMG.

The impact of fatigue on MG has not been studied widely despite its substantial impact on patients’ lives. The effect of other complement C5 inhibitors, eculizumab and ravulizumab, on fatigue in their Phase 3 and OLE studies was assessed using the raw scores on an alternative, longer instrument—the 19-item Neuro-QoL fatigue subscale—and variable degrees of reduction from baseline were reported, highlighting that complement C5 inhibitors can reduce fatigue in patients with MG [28–30]. However, zilucoplan is the first C5 inhibitor to show a clinically meaningful reduction in fatigue. To conclude, daily subcutaneous injections of the complement C5 inhibitor zilucoplan led to rapid and clinically meaningful improvements in fatigue that were sustained for more than a year in patients with gMG. The data showed rapid benefits in fatigue for patients receiving zilucoplan, with sustained and meaningful improvements across all baseline fatigue severity levels, including severe fatigue where the effect is especially debilitating for patients. The majority of patients reported no or mild fatigue at Week 60, improving from severe or moderate fatigue at baseline. These data highlight that treatment with zilucoplan can result in a sustained reduction in fatigue, a symptom known to be particularly debilitating for patients with gMG.

### Supplementary Information

Below is the link to the electronic supplementary material.

Supplementary file1 (DOCX 68 KB)

## Data Availability

Underlying data from this manuscript may be requested by qualified researchers 6 months after product approval in the USA and/or Europe or global development is discontinued, and 18 months after trial completion. Investigators may request access to anonymised individual patient-level data and redacted trial documents, which may include analysis-ready datasets, the study protocol, annotated case report forms, statistical analysis plans, dataset specifications, and the clinical study report. Prior to use of the data, proposals need to be approved by an independent review panel at www.Vivli.org and a signed data-sharing agreement will need to be executed. All documents are available in English only, for a pre-specified time, typically 12 months, on a password-protected portal.
