# Supplementary material for: Improvement of fatigue in generalised myasthenia gravis with zilucoplan
Source: J Neurol. 2024 Feb 24;271(5):2758–67. doi: 10.1007/s00415-024-12209-3 (PMC11055786; doi:10.1007/s00415-024-12209-3)
Supplement: Supplementary file 1 — Supplementary file1 (DOCX 68 KB) [file 415_2024_12209_MOESM1_ESM.docx]

Improvement of fatigue in generalised myasthenia gravis with zilucoplan

**Michael D. Weiss^1^, Miriam Freimer^2^, M. Isabel Leite^3^, Angelina Maniaol^4^, Kimiaki Utsugisawa^5^, Jos Bloemers^6^, Babak Boroojerdi^7^, Emily Howard^8*^, Natasa Savic^9^, James F. Howard Jr^10^**

^1^Department of Neurology, University of Washington Medical Center, Seattle, WA, USA; ^2^Department of Neurology, The Ohio State University Wexner Medical Center, Columbus, OH, USA; ^3^Nuffield Department of Clinical Neurosciences, University of Oxford, Oxford, UK; ^4^Department of Neurology, Oslo University Hospital, Oslo, Norway; ^5^Department of Neurology, Hanamaki General Hospital, Hanamaki, Japan; ^6^UCB Pharma, Brussels, Belgium; ^7^UCB Pharma, Monheim, Germany; ^8^UCB Pharma, Slough, UK and Cogent Skills^*^, Warrington, UK; ^9^UCB Pharma, Bulle, Switzerland; ^10^Department of Neurology, The University of North Carolina at Chapel Hill, Chapel Hill, NC, USA

^*^Currently working at the University of Bath, Bath, UK

Journal: Journal of Neurology

Corresponding author: Dr Michael D. Weiss

Address: Department of Neurology, University of Washington Medical Center, 1959 NE Pacific Way, Seattle, WA 98195, USA

Tel: 1-206-598-7688

Email: [mdweiss@uw.edu](mailto:mdweiss@uw.edu)

**Table of contents**

[Supplementary appendix 3](#_Toc153788952)

[Supplementary Text 1 Plain language summary 3](#_Toc153788953)

[Supplementary Text 2 MG-ADL, QMG and MG-QoL 15r assessments 4](#_Toc153788954)

[Supplementary Table 1 Known-groups validity analysis with MGFA disease classification at screening 5](#_Toc153788955)

# Supplementary appendix

### Supplementary Text 1 Plain language summary

Fatigue is a troublesome symptom for people with myasthenia gravis (MG) and has a severe impact on their quality of life, but it is not measured during routine clinical visits. In a Phase 3 clinical trial (RAISE), zilucoplan significantly improved MG symptoms with few side effects. This study aimed to understand how patients’ fatigue is affected by long-term use of zilucoplan. The study was run in two parts: the 12-week RAISE study, in which patients received either zilucoplan or placebo, and the ongoing, long-term RAISE-XT study, in which all patients who completed RAISE could receive zilucoplan. We used the eight-point Quality of Life in Neurological Disorders (Neuro-QoL) Short Form fatigue questionnaire, which was completed by the patients to assess their feelings of exhaustion, frustration at being unable to do tasks because of their fatigue, and limitations to social activity. Further analysis of the questionnaire responses allows us to determine levels of fatigue severity, classified as none (“no problems”), “mild problems”, “moderate problems”, and “severe problems”.

During RAISE, 86 patients received zilucoplan and 88 received placebo, and all patients who completed RAISE entered the RAISE-XT study to receive zilucoplan. Patients receiving zilucoplan in RAISE had a marked reduction in their fatigue at the end of RAISE (after 12 weeks of zilucoplan treatment), which was sustained up to Week 60. Also, at the beginning of the study, three-quarters of the patients had “severe” or “moderate” fatigue. By Week 12, nearly half of the patients receiving zilucoplan moved to a less severe fatigue level. At Week 60, about two-thirds of patients either had no fatigue at all, or said their fatigue was “mild”. Our study highlights that treatment with zilucoplan can result in a lasting and meaningful reduction in fatigue for people with generalised MG.

### Supplementary Text 2 MG-ADL, QMG and MG-QoL 15r assessments

MG-ADL, QMG and MG-QoL 15r scores were assessed at Weeks 1, 2, 4, 8 and 12 in the double-blind period and Week 13 (Week E1), Week 14 (Week E2), Week 16 (Week E4), Week 20 (Week E8) and Week 24 (Week E12) in the open-label extension study. After Week 24, these scores were assessed at quarterly visits. Change from baseline up to Week 60 in MG-ADL, QMG and MG-QoL 15r scores was estimated using a linear mixed model repeated measures analysis of covariance, with baseline MG-ADL score, baseline QMG score, baseline score (for MG-QoL 15r only), geographical region, qualifying study factor, visit and baseline score X visit (interaction term) as fixed effects, and participant as a random effect using an unstructured correlation structure.

E, extension; MG-ADL, Myasthenia Gravis Activities of Daily Living; MG-QoL 15r, Myasthenia Gravis Quality of Life 15-item revised; QMG, Quantitative Myasthenia Gravis.

### Supplementary Table 1 Known-groups validity analysis with MGFA disease classification at screening

| **Known-group (level)** | **Statistics** | **Baseline Neuro-QoL Short Form Fatigue T-score** |
| --- | --- | --- |
| **MGFA Disease Class II (n=46)** | Mean (SD) | 56.31 (7.87) |
|  | Median | 57.60 |
|  | Min, Max | 34.10, 74.10 |
| **MGFA Disease Class III/IV (n=115)^a^** | Mean (SD) | 58.47 (8.52) |
|  | Median | 59.90 |
|  | Min, Max | 29.50, 74.10 |
| **MGFA Disease Class II vs III/IV** | Difference in mean (95% CI)^b^ | −2.16 (−5.03, 0.71) |
|  | Effect size^c^ | −0.26 |
|  | p-value (F-test) | 0.140 |
|  | p-value (Kruskal-Wallis test) | 0.092 |

^a^Level “MGFA Disease Class IV” had a very small sample size (n<15) and hence was collapsed into the adjacent level “Class III” based on clinical reference. ^b^All differences in means are calculated relative to the previous known-group; 95% CI estimated from the t-test. ^c^Effect size is estimated as the difference in means divided by the pooled SD of the total score at baseline.

CI, confidence interval; MGFA, Myasthenia Gravis Foundation of America; Neuro-QoL, Quality of Life in Neurological Disorders; SD, standard deviation.
